# Supplementary material for: Intramale variation in sperm size: functional significance in a polygynous mammal
Source: PeerJ. 2015 Dec 8;3:e1478. doi: 10.7717/peerj.1478 (PMC4675104; doi:10.7717/peerj.1478)
Supplement: Table S1 — Significant relationships are shown in bold type. P value is shown in brackets. CV, intramale coefficient of variation; PCA, principal component analysis; TSL, total sperm length; HW, head width; HL, head length; HA, head area; HP, head perimeter; HL/HW, head ellipticity; FL, flagellum length; MPL, midpiece length; PPL, principal plus terminal piece length; VAP, average path velocity; VCL, curvilinear velocity; VSL, straight linear velocity. [file peerj-03-1478-s001.docx]

|  | **Absolute testes mass (log_10_)** | **Sperm velocity (PCA)** | **VAP** | **VCL** | **VSL** | **Progressive sperm motility** | **Normal morphology** | **Sperm number** |
| --- | --- | --- | --- | --- | --- | --- | --- | --- |
| TSL (CV) | **-0.58 (0.016)** | **-0.54 (0.025)** | **-0.56 (0.021)** | -0.14 (0.594) | **-0.56 (0.018)** | **-0.49 (0.045)** | -0.28 (0.280) | -0.01 (0.991) |
| HW (CV) | **-0.70 (0.002)** | 0.01 (0.979) | 0.04 (0.874) | -0.07 (0.804) | 0.013 (0.960) | -0.06 (0.832) | -0.27 (0.290) | -0.09 (0.733) |
| HL (CV) | -0.12 (0.658) | -0.37 (0.139) | -0.37 (0.144) | 0.02 (0.948) | **-0.49 (0.048)** | -0.48 (0.050) | -0.40 (0.114) | 0.14 (0.588) |
| HA (CV) | -0.48 (0.050) | 0.14 (0.599) | 0.14 (0.591) | 0.07 (0.784) | 0.12 (0.648) | 0.09 (0.725) | **-0.54 (0.027)** | -0.16 (0.554) |
| HP (CV) | -0.27 (0.298) | -0.04 (0.869) | -0.05 (0.865) | 0.07 (0.786) | -0.10 (0.699) | -0.11 (0.688) | **-0.53 (0.029)** | -0.05 (0.842) |
| HL/HW (CV) | -0.36 (0.162) | **-0.49 (0.047)** | -0.45 (0.069) | -0.12 (0.662) | **-0.57 (0.016)** | **-0.63 (0.007)** | -0.08 (0.767) | 0.22 (0.392) |
| FL (CV) | -0.47 (0.056) | **-0.61 (0.010)** | **-0.62 (0.008)** | -0.14 (0.588) | **-0.65 (0.005)** | **-0.57 (0.016)** | -0.19 (0.466) | 0.04 (0.872) |
| MPL (CV) | **-0.49 (0.046)** | -0.26 (0.306) | -0.29 (0.267) | -0.03 (0.899) | -0.28 (0.272) | -0.15 (0.579) | -0.17 (0.512) | -0.30 (0.241) |
| PPL (CV) | -0.33 (0.193) | **-0.61 (0.009)** | **-0.64 (0.006)** | -0.07 (0.795) | **-0.69 (0.002)** | **-0.63 (0.007)** | -0.08 (0.769) | 0.19 (0.476) |

**Table S1.** Relationships of the intramale CV of sperm morphometry parameters with absolute testes mass and sperm traits in red deer.

Significant relationships are shown in bold type. *P* value is shown in brackets. CV= intramale coefficient of variation; PCA= principal component analysis; TSL= total sperm length; HW= head width; HL= head length; HA= head area; HP= head perimeter; HL/HW= head ellipticity; FL=flagellum length; MPL= midpiece length; PPL= principal plus terminal piece length; VAP= average path velocity; VCL= curvilinear velocity; VSL= straight linear velocity.
